# Editorial: Veterinary public health: veterinary medicine's current challenges in a globalised world

**DOI:** 10.3389/fvets.2025.1564614

**Published:** 2025-03-10

**Authors:** Reinhard Fries, Diana Meemken, Ivan Nastasijevic, Suporn Thongyuan

**Affiliations:** ^1^Free University of Berlin, Berlin, Germany; ^2^Institute of Meat Hygiene and Technology, Belgrade, Serbia; ^3^Department of Veterinary Public Health, Faculty of Veterinary Medicine, Kasetsart University, Nakhon Pathom, Thailand

**Keywords:** veterinary public health, one health, zoonotic agents, veterinary training, editorial

## 1 Introduction

The transfer of zoonotic agents is a normal and well-known phenomenon. Every substance or living thing is a potential carrier, while a favorable environment increases the risk of transmission. As sources of zoonotic agents, all types of domestic animals are also in focus, whether working animals (including those used for sport), companion animals or pets, or animals reared for food production. The keeping or rearing of these animals is not uniform worldwide, as local culture plays a major role in local approaches to animals. Also, stray animals pose a global challenge in terms of their welfare and their role in the transmission of zoonoses.

Veterinary medicine (VM) has a major responsibility in preventing zoonosis transmission, with two main branches involved: clinical VM and preventive veterinary public health (VPH). Geographically, the profile of the veterinary profession is by no means uniform, as lifestyles in industrialized and developing countries differ. In developed countries, VM is frequently focused on companion animals, sometimes with tools and equipment close to those used in human medicine. Hence, the treatment of companion animals is highly regarded by the general public and the veterinary profession itself.

On the other hand, VPH is frequently associated with administration. However, VPH is much broader in scope than just an administrative service, as it deals with population and preventive medicine, progressive, modern food safety and animal welfare. VPH is responsible for the entire local animal population and must be adapted and adaptable to changing regional circumstances. Both interventions and solutions depend on the individual disease situation, and for successful measures, VPH is now based on complex horizontal, interdisciplinary solutions. Therefore, it is misleading to describe VPH only as a domain of legislation and administration. It is true that national and international legislation is the basis for appropriate structures and intervention procedures. Building on this, however, VPH provides the capacity and interdisciplinary competence for intervention to keep a risk situation under control.

Local events sometimes have consequences on a broader geographical scale, reaching global importance. Today, information technology (IT) serves as a tool for immediate communication. For the first time in human history, with the help of IT, information about a disease now travels faster than the agent, promising good options for successful VPH interventions.

## 2 This Research Topic's focus

Contributions to this Research Topic come from the global south and north. Some contributions point to a lack of practical realization of ideas, e.g., animal agency (Ameli and Krämer) and antibiotic stewardship (Gunn-Sandell et al.), while others point to gaps in practical performance, e.g., the One-Health concept, which has been discussed for years, but still lacks practical implementation.

The contributions generally show that personal issues within the VPH profession are considered a major point of research. Indeed, VPH curricula in universities are a central means for the future development of concepts for our profession and a better understanding of VPH. Unfortunately, in industrialized countries, there is a lack of student interest in VPH, perhaps because of the safety of life in such countries. However, all VM students must learn the full scope of VPH, such as understanding the background and purpose of legislative interventions. Such in-depth knowledge is required for the smooth acceptance and implementation of legislation. Moreover, study courses for both undergraduates and postgraduates need to better utilize IT as an excellent tool for education and training on global VPH concerns. Finally, the One-Health concept must be established in the curricula of both human and veterinary medicine.

### 2.1 Animal welfare

Depending on geography and history, our attitudes toward animals can differ; call it the ethical line of society toward animals. Two papers discuss the animal welfare sector, raising the question of how we treat laboratory animals, stray dogs and stray cats. In the case of laboratory animals, Ameli and Krämer questioned the culture of care for them in Germany. The authors asked different experts from institutions across Germany about their position (regulators, managers, scientists, care persons) and received different answers, ranging from a high level of responsibility (care persons) to formal aspects (regulators), and awareness of the need to provide animal welfare (scientists). Managers emphasized the culture of care to a lesser extent.

To keep stray dogs and cats under control, an Ethical Population Management Program is in use on a university campus in Minas Gerais, Brazil. Bicalho et al. studied its efficacy and perceptions of campus users. The measures taken in the programme were widely endorsed.

### 2.2 Personnel and administrative issues

Diseases do not recognize borders, so contact between neighbors is a basic condition for successful veterinary measures. Auplish et al. analyzed the capability of veterinary field staff in Vietnam for disease prevention, preparedness and intervention. Vertically, training and competence were more limited at the district and commune level than at the national level. However, inequities were also found at the local level (horizontally).

The World Organization for Animal Health (WOAH) has compiled a list of competencies that veterinarians need in order to support national veterinary services, and Ethiopia has consequently implemented a new national curriculum. Bessler et al. identified barriers to its practical implementation: i.e., organization of veterinary services, inspection and certification procedures, practical application of the regulatory framework for disease prevention and control, lack of teaching and training materials and financial constraints.

Nyokabi, Phelan, et al. addressed infection/zoonosis control and biosecurity measures among VM students in Ethiopia. VM students were aware of the public health risks posed by zoonoses and of the important role of cooperation between human and veterinary medicine. However, the students showed poor knowledge of infection control measures and biosecurity or even measures to reduce occupational risks. The authors stressed the importance of students' access to information about the risks of zoonoses, infection control and biosecurity measures, which could also affect the way veterinarians themselves behave in the event of a zoonotic disease.

In this sense, Hoet et al. presented guidance for VM educational institutions to improve curricula in VPH and population medicine. Students should be able to respond to the challenges from day one of their professional life. The blueprint was a standard curriculum developed by the WOAH (see also Bessler et al.).

Nyokabi, Wood, et al. investigated veterinarians' knowledge and VPH competence in relation to cattle disease symptoms in Ethiopia. They were able to identify such diseases, but the authors found gaps when it came to the consequences in the field of VPH.

The general issue of personal pressure during one's occupational life should always be taken into consideration. Neubauer et al. surveyed all registered veterinarians and VM students in Austria. Administration, animal suffering and different forms of communication with animal owners were the most burdensome pressures for working veterinarians. VM students were already aware of the coming occupational stress; however, they anticipated other stressors than those reported by their working colleagues.

### 2.3 Antimicrobial resistance

For many years, antimicrobial resistance has been a major point of interest within VPH. Lekagul et al. described the Voluntary Optimization of Antimicrobial Consumption (VOAC) programme in Thailand. Antimicrobial resistance concerns not only food animals, as resistance can also start from companion animals. Gunn-Sandell et al. investigated the knowledge of antimicrobial drug use and resistance among veterinary clinic support staff. The authors presented an Antibiotic Stewardship Program, i.e., protocols, programmes and other materials that promote appropriate antibiotic use. Antimicrobial resistance was well-known, while antimicrobial stewardship was underestimated.

### 2.4 Surveillance of food animals

In addition to traditional meat inspection, which was established a long time ago, the whole food chain approach to meat production was triggered by the BSE disaster in the 1990s. This modern approach is now well established and provides information on the history of an animal or a herd. However, when such information is unavailable, inspection procedures for food animals depend on local circumstances and specific situations. Bekele Atoma et al. conducted a study on small ruminants in the Ethiopian Highlands, where food chain information was not available. Therefore, traditional inspection at the abattoir was applied. The authors concluded that the reduction of the parasite burden and improved handling could increase the profitability of the small ruminant meat sector in Ethiopia.

